# Grundprinzipien der operativen Versorgung der distalen Radiusfraktur

**DOI:** 10.1007/s00113-024-01429-x

**Published:** 2024-04-23

**Authors:** Ulrike Seeher, Simone Bode, Rohit Arora

**Affiliations:** Universitätsklinik für Orthopädie und Traumatologie, Medizinische Universität Innsbruck, Anichstr. 35, 6020 Innsbruck, Österreich

**Keywords:** Intraartikuläre Frakturen, Palmare Plattenosteosynthese, Frakturfixierung, Arthroskopie, Komplikation, Intra-articular fractures, Volar plating, Fracture fixation, Arthroscopy, Complication

## Abstract

Die distale Radiusfraktur ist eine der häufigsten Frakturen der oberen Extremität. Nach entsprechender Diagnostik mithilfe von nativradiologischen und meist computertomographischen bildgebenden Untersuchungen fällt die Entscheidung zur konservativen oder zur operativen Therapie. Ist die Indikation zur operativen Versorgung gegeben, stehen diverse Möglichkeiten der Reposition und Fixation zur Verfügung. Das Spektrum reicht von geschlossenen über offene Verfahren bis hin zur unterstützenden begleitenden Arthroskopie. Eine entsprechende präoperative Aufklärung der PatientInnen über den Eingriff sowie die geplante Nachbehandlung ist essenziell. Ziel der Versorgung ist die Wiederherstellung der Handgelenkfunktion unter Erhalt von Beweglichkeit und Kraft bei niedrigem Komplikationsrisiko. Allen operativen Verfahren ist das Prinzip der Reposition zur Wiederherstellung der anatomischen Verhältnisse und anschließender Fixation gemeinsam. Als geschlossene Verfahren stehen die Fixation mit Kirschner-Drähten sowie der Aufbau eines Fixateur externe zur Verfügung. Die palmare winkelstabile Plattenosteosynthese hat sich in den letzten Jahren als Methode der Wahl für einen Großteil der zu versorgenden Frakturen etabliert. Für spezielle Frakturmuster und zur Behandlung von Begleitverletzungen kann eine arthroskopische Unterstützung indiziert sein. Ein einheitlicher Konsensus über die beste Verfahrenswahl besteht nicht. In diesem Beitrag werden die möglichen Verfahren, einschließlich ihrer Zugänge, Fixationstechniken und spezifischer Nachbehandlung, beleuchtet.

## Abstract (en)

Distal radius fractures are one of the most frequent fractures of the upper extremities. The decision for conservative or surgical treatment is made after appropriate diagnostics using conventional radiographic and usually computed tomography imaging examinations. If the indications for surgical treatment are present, various options for reduction and fixation are available. The spectrum ranges from closed to open procedures up to accompanying arthroscopic support. Appropriate preoperative patient education about the procedure and the planned postinterventional treatment is essential. The goal of treatment is to restore wrist function while maintaining mobility and strength with a low risk of complications. All surgical procedures share the principle of reduction to restore anatomical relationship followed by fixation. Closed procedures include fixation with Kirschner wires and the construction of an external fixator. Volar locking plate osteosynthesis has become established in recent years as the method of choice for the majority of the fractures to be treated. For special fracture patterns and the treatment of accompanying injuries, arthroscopic support can be indicated. There is no uniform consensus on the best choice of procedure. This article discusses the possible procedures including the approaches, fixation techniques and specific follow-up treatment.

Distale Radiusfrakturen, die zu den häufigsten Frakturen zählen, können erhebliche Auswirkungen auf die Funktionalität und die Lebensqualität der Betroffenen haben, da der distale Radius eine entscheidende Rolle in der komplexen Biomechanik des Handgelenks und der Hand spielt. Eine adäquate und zielgerichtete Behandlung ist unerlässlich. Betroffen sind v. a. 2 verschiedene PatientInnengruppen mit zweigipfliger Altersverteilung: junge, sportlich aktive und häufig männliche Patienten aufgrund von Hochenergietraumata sowie ältere PatientInnen mit oft postmenopausaler Osteoporose aufgrund von Niedrigenergietraumata [36]. Im Folgenden wird auf die operativen Grundprinzipien zur Frakturversorgung eingegangen.

## Diagnostik

Am Beginn steht die fokussierte Anamnese, wobei insbesondere Unfallhergang, relevante Begleiterkrankungen wie z. B. Osteoporose sowie funktioneller Anspruch der PatientInnen durch berufliche und Freizeitaktivität Beachtung finden sollten.

Bei der körperlichen Untersuchung muss neben der Beurteilung der Haut-Weichteil-Situation besonderes Augenmerk auf die Überprüfung der peripheren Durchblutung, Motorik und Sensibilität (prDMS) gelegt werden, um ein traumatisches Karpaltunnelsyndrom mit Notwendigkeit zur akuten Intervention zu detektieren.

Zur Detektion eines Karpaltunnelsyndroms sollte der pDMS-Kontrolle besonderes Augenmerk zukommen

**Figure 1:**
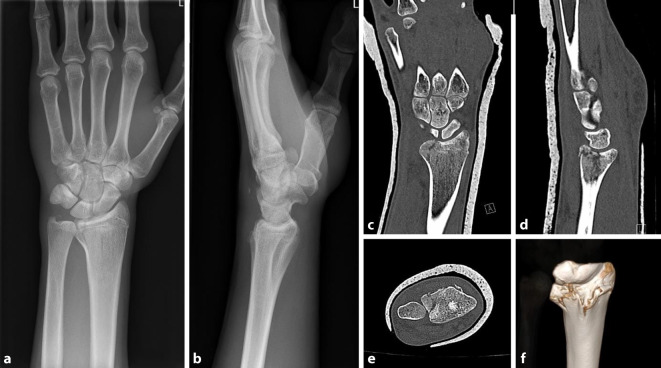

Als bildgebende Untersuchungen sind die konventionellen Röntgenaufnahmen im p.-a.- sowie im seitlichem Strahlengang Standard. Ergänzend sollte bei Verdacht auf eine intraartikuläre Beteiligung eine CT-Untersuchung erfolgen. Eine 3D-Rekonstruktion ist bei intraartikulären Frakturen anzustreben (Abb. 1). Die MRT-Bildgebung hat in der Akutsituation wegen der Möglichkeit der intraoperativen Arthroskopie nahezu keinen Stellenwert [3, 11].

## Operative Therapie

### Indikationen

Die Indikationsstellung zur operativen Versorgung bedarf der Berücksichtigung einer Vielzahl von Faktoren wie Unfallmechanismus, Frakturmuster, Weichteilverhältnissen, Begleitverletzungen und funktionellem Bedarf. Operationsindikationen ergeben sich aus den in Tab. 1 zusammengefassten Faktoren.

**Table 1:** 

Klinische Kriterien	Offene Frakturen der Typen II und III nach Gustilo und Anderson [12]
	Gefäß- und Nervenläsion, insbesondere traumatische Kompression des N. medianus
	Geschlossene Frakturen mit Weichteilverletzung der Grade II und III nach Tscherne und Oestern [33]
Radiologische Kriterien	Radialer Höhenverlust > 2 mm
	Radioulnarer Inklinationswinkel < 10°
	Verlust der Palmarkippung > 10°
	Verlust der Dorsalkippung > 20°
	Gelenkstufe > 2 mm
	Inkongruenz im DRUG > 1 mm oder radioulnare Dissoziation
	Repositionsverlust im Verlauf
	Fraktur der palmaren und dorsalen Gelenkkante (Barton- und Reversed-Barton-Fraktur)

Abkürzung s. Abkürzungsverzeichnis

Zu berücksichtigen sind jedoch auch relative Indikationen wie Mehrfachverletzungen, wie sie bei Serienverletzungen derselben Extremität oder Verletzungen der Gegenseite vorkommen, sowie der PatientInnenwunsch, das biologische Alter des Patienten und sein funktioneller Anspruch, z. B. aufgrund des Berufs [20, 22].

### Ziele

Die grundsätzlichen Behandlungsziele der Frakturbehandlung sind Frakturheilung, Schmerzfreiheit, Wiederherstellung der Handgelenkfunktion, Erhalt von Beweglichkeit und Kraft sowie die erfolgreiche Wiedereingliederung in das soziale Umfeld. Zusätzlich bestehen folgende Anforderungen an die operative Versorgung: Wiederherstellung der anatomischen Verhältnisse, nach Möglichkeit frühfunktionelle Behandlung, adäquate Adressierung von Begleitverletzungen und ein geringes Komplikationsrisiko. Dies kann nur durch das Zusammenspiel aus entsprechender präoperativer Vorbereitung, operativer Umsetzung mithilfe der etablierten Methoden und postoperativer Versorgung, einschließlich ergotherapeutischer Beübung, erreicht werden.

### PatientInnenaufklärung

Die PatientInnenaufklärung umfasst neben den allgemeinen Operationsrisiken wie Blutung, Infektion, Wundheilungsstörung, Narbenbildung und allergischen Reaktionen spezielle Risiken der geplanten Versorgung ([4]; Tab. 2).

**Table 2:** 

Entwicklung eines CRPS
Verbleibendes Bewegungs- und Funktionsdefizit
Beuge- und Strecksehnenruptur, insbesondere der FPL-Sehne
Nervenverletzung: Medianusverletzung mit Schädigung des Thenarasts oder Läsion des R. palmaris; R. superficialis n. radialis mit entsprechendem motorischen und sensiblen Defizit
Posttraumatisches Kompartmentsyndrom
(Folgeeingriffe mit Implantatentfernung)
(Entnahme eines autogenen Knochentransplantats/Einbringung eines allogenen Knochentransplantats)

Abkürzungen s. Abkürzungsverzeichnis

Des Weiteren sollten die PatientInnen bereits im Aufklärungsgespräch über die geplante Nachbehandlung, insbesondere Dauer der Ruhigstellung, voraussichtliche Wiederaufnahme der Alltags- und beruflichen Aktivität sowie notwendige Therapien informiert werden, um eine adäquate postoperative Versorgung zu gewährleisten.

### Fixationsmöglichkeiten

#### Kirschner-Draht

Eine K‑Draht-Fixation als alleinige und endgültige Versorgungsmethode wird in der Universitätsklinik für Orthopädie und Traumatologie der Medizinischen Universität Innsbruck lediglich im Kindes- und im Jugendalter durchgeführt. Es erfolgen die geschlossene Reposition und perkutane Fixation mithilfe von 2 bis 3 K-Drähten der Stärken 1,2–1,6 mm mit langem Gewinde, um einen sicheren Halt in der Gegenkortikalis zu garantieren. Zur Verfügung stehen die Methoden nach Kapandji [10] mithilfe der intrafokalen Drahteinbringung sowie die Methode nach Willenegger [37] mithilfe der extrafokalen Drahteinbringung.

Die häufigsten Komplikationen sind durch eine ungenügende Präparation der Weichteile bedingt

Die Stifte sollten über Hautniveau umgebogen und gekürzt werden; dies ermöglicht die einfache Entfernung nach der Frakturheilung ohne Notwendigkeit einer erneuten Anästhesie. Ein vermehrtes Infektionsrisiko wird kontrovers diskutiert, in einer rezenten Studie jedoch nicht belegt [1].

Nachfolgend wird eine palmare Unterarmgipslonguette angelegt, die je nach PatientInnenalter 4 bis 6 Wochen getragen wird. Ab sofort kann eine ergotherapeutische Beübung der Finger, nicht jedoch des Handgelenks erfolgen, um eine sekundäre Dislokation der Stifte zu vermeiden. An die Gipsabnahme schließen sich die funktionelle Weiterbehandlung und nach dem Ende der 6. Woche der Belastungsaufbau an. Acht Wochen nach der CRIF können bei guter Konsolidierung die Stifte in Lokalanästhesie entfernt werden. Empfohlen werden regelmäßige Röntgenkontrollen 10 bis 14 Tage sowie 6 Wochen postoperativ. Die häufigsten Komplikationen entstehen durch eine ungenügende Präparation der Weichteile, die zu Nerven- und/oder Sehnenverletzungen führen können. Fehlt die kortikale Abstützung, besteht ein erhöhtes Risiko der sekundären Dislokation. Bei über die Haut hinausstehenden Stiften besteht die Gefahr einer Infektion an der Stifteintrittsstelle [14, 25].

#### Schraubenosteosynthese

Die häufigste Indikation für eine isolierte Schraubenosteosynthese ist die Fraktur des Processus styloideus radii (Chauffeur-Fraktur). Nach offener Reposition wird die Fraktur perkutan mithilfe einer kanülierten Schraube der Durchmesser 2,7–3,2 mm und bei Bedarf mithilfe eines K‑Drahts als Derotationsstift fixiert.

Diese Versorgung ermöglicht eine sofortige funktionelle Nachbehandlung; eine Ruhigstellung ist nur bei begleitendem karpalen Bandschaden notwendig. Der Derotationsstift kann nach 6 Wochen entfernt werden, die kanülierte Schraube wird nur bei lokaler Irritation entfernt. Auch hier besteht die Hauptgefahr durch mangelnde Präparation der Stifteintrittsstellen; bei bestehender Kompressionszone kann es zum Nachsinken der Fraktur kommen [25].

#### Fixateur externe

Diese Versorgung ist hochgradig instabilen Frakturen mit ausgedehntem Weichteilschaden und im Rahmen eines Polytraumas vorbehalten. Zum Aufbau eines radiometakarpalen Fixateurs externe werden je 2 Pins der Stärken 3–4 mm in die Radiusdiaphyse sowie in die Diaphyse des 2. Metakarpalknochens eingebracht. Die Pin-Insertionsstelle liegt am Radius posterolateral im mittleren Drittel der Radiusdiaphyse. Nach dem Aufbau des Verbindungsstabs erfolgt die definitive Reposition; diese wird über eine leichte Palmarflexion und Ulnarduktion erreicht. Der Fixateur wird für insgesamt 6 Wochen belassen. Alternativ ist eine sekundäre ORIF möglich. Hauptkomplikationen sind die Infektion der Pin-Eintrittsstellen, Frakturen des 2. Mittelhandknochens und Verletzungen des R. superficialis n. radialis [26, 29].

#### Palmare winkelstabile Plattenosteosynthese

Der operative Zugang zum distalen Radius erfolgt zumeist über den modifizierten Henry-Zugang [15]. An die Darstellung der Fraktur und Präparation des Plattenlagers schließt sich die Reposition der Fraktur an; diese kann temporär mithilfe von K‑Drähten retiniert werden. Nach Überprüfung der Reposition mithilfe des C‑Bogens wird die Platte eingebracht. Anzustreben ist eine Plattenlage proximal der Watershed-Linie, um das Risiko für Beugesehnenirritationen, insbesondere der FPL-Sehne, zu minimieren [31]. Die Platte wird zunächst über das Gleitloch fixiert, sodass eine Positionskorrektur nach proximal oder distal möglich bleibt. Zunächst wird die distale Schraubenreihe, die hauptsächlich der Abstützung des zentralen subkortikalen Knochens dient, besetzt. Nachfolgend erfolgt das Besetzen der proximalen Reihe zur zusätzlichen Abstützung des dorsalen subchondralen Knochens. Durch diese doppelreihige Abstützung kann das Risiko eines sekundären Repositionsverlusts reduziert werden [19]. Nach abschließender bildgebender Kontrolle können, wenn möglich, eine Refixation des M. pronator quadratus zur Bedeckung der Platte und Reduktion von Sehnenirritationen durchgeführt werden; ein eindeutiger Vorteil dieses Vorgehens ist nicht belegt [23].

Anzustreben ist eine Plattenlage proximal der Watershed-Linie

**Figure 2:**
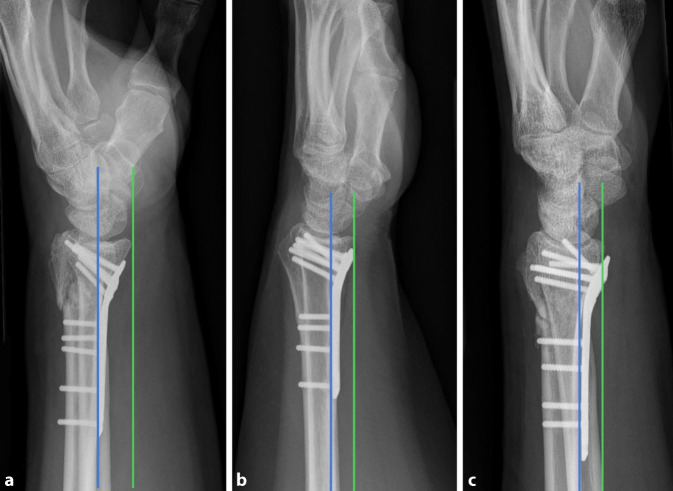

Aufgrund der Plattenlage kann es zu Irritationen bis hin zur Ruptur der Beugesehnen, insbesondere der FPL-Sehne, kommen. Zur Einschätzung der Rupturgefahr der Beugesehnen sollte sich in nachfolgenden Röntgenkontrollen an der Soong-Klassifikation orientiert werden ([16, 31]; Abb. 2).

Bei ungünstiger Plattenlage ist eine frühzeitige Metallentfernung nach der Frakturkonsolidierung empfohlen [9]. Hingegen sind durch überlange Schrauben auf der Dorsalseite die Strecksehnen gefährdet, daher sollte intraoperativ eine Skyline-View-Aufnahme erstellt werden, um einen möglichen Schraubenüberstand zu detektieren ([32]; Abb. 6h,i). Insbesondere bei osteoporotischem Knochen besteht das Risiko eines sekundären Repositionsverlustes, dem durch korrekte subchondrale Schraubenpositionierung entgegengewirkt werden kann [18]. Des Weiteren kann ein postoperatives Karpaltunnelsyndrom oder ein CRPS entstehen.

#### Dorsale winkelstabile Plattenosteosynthese

Besonders geeignet ist der dorsale Zugang, um dorsoulnare Kantenfragmente anatomisch zu reponieren und fixieren. Dies erfordert die Mobilisation zumindest des 2., 3. und 4. Strecksehnenfachs. Oft ist es zusätzlich notwendig, einen Teil des Tuberculum Listeri abzutragen, um eine anatomische Plattenlage zu gewährleisten. Je nach gewähltem Plattendesign wird eine Abstützung sowohl der radialen als auch der intermediären Säule nach dem Dreisäulenmodell von Rikli [27] ermöglicht. Nach der anatomischen Reposition erfolgt die temporäre Fixation mithilfe von K‑Drähten; die definitive Versorgung kann entweder mithilfe einer Doppelplattenosteosynthese oder einer zweischenkligen Gliederplatte (Phi-Platte) durchgeführt werden [30]. Begleitend kann eine Neurotomie des N. interosseus posterior erwogen werden [15]. Zur Reduktion von Strecksehnenirritationen werden eine Retinakulumplastik und die Implantatentfernung nach knöcherner Konsolidierung empfohlen [35].

### Arthroskopie

Die Indikationen zur Arthroskopie sind Tab. 3 aufgeführt.

**Table 3:** 

Sagittale Frakturlinie auf Höhe des SL-Spalts
Sagittale oder frontale Frakturlinie mit großen Fragmenten (Die-Punch-Fragment)
Impaktiertes zentrales Fragment
Instabiles DRUG
SL-Spalt-Erweiterung

Abkürzungen s. Abkürzungsverzeichnis

Zumeist erfolgt die Arthroskopie in Kombination mit einer palmaren Plattenosteosynthese. Nach der Positionierung der Platte wird die Hand des Patienten im Mädchenfänger aufgehängt und eine leichte Traktion mit 2–4 kg Gewicht appliziert. Der Zugang erfolgt zunächst über die Portale 3/4 sowie 6R mithilfe eines kleinkalibrigen Arthroskops (2,7 mm). Ist eine Beurteilung der midkarpalen Strukturen gewünscht, werden zusätzlich das radiale und ulnare midkarpale Portal benötigt. Empfohlen wird die trockene Arthroskopie, um eine Weichteilschwellung und das Risiko eines Kompartmentsyndroms zu reduzieren. Nun kann die Frakturreposition überprüft und ggf. optimiert werden („fine tuning“); eine intraartikuläre Schraubenlage kann ausgeschlossen werden. Hochgradige SL-Band-Verletzungen (Geissler-Stadium IV [8]) werden mithilfe der offenen dorsalen Bandrefixation mit einem Anker und zusätzlicher temporärer Transfixation versorgt. Läsionen des TFCC werden detektiert; auf die detaillierte Versorgung von Begleitverletzungen wird im Beitrag von Wieschollek und Megerle im gleichen Themenheft eingegangen, weshalb im vorliegenden Beitrag auf eine weitere Beschreibung verzichtet wird [2, 17].

### Osteoporotische Trümmerfrakturen bei älteren PatientInnen

**Figure 3:**
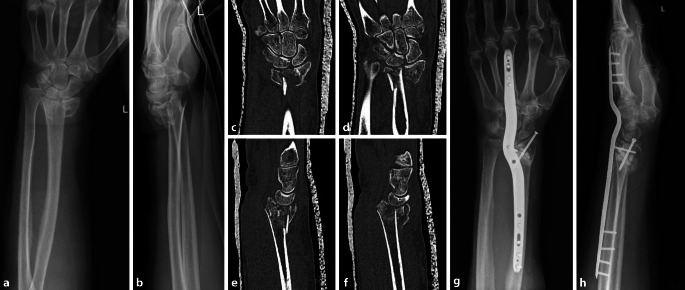

Haben PatientInnen trotz höheren Alters einen funktionellen (zur Erfüllung der Alltagsaktivitäten, Sport) oder auch kosmetischen Bedarf bezüglich ihres Handgelenks, kann eine Entscheidung zur operativen Therapie gefällt werden. In den meisten Fällen sind eine anatomische Rekonstruktion und Plattenosteosynthese aufgrund der meist vorbestehenden Trümmerfraktur, des metaphysären Knochendefekts und der osteoporotischen Knochenqualität nicht mehr möglich. Hierfür stehen u. a. Spanning-Platten zur Verfügung, wobei eine handgelenküberbrückende dorsale Platte von der Radiusmetaphyse zum 2. oder 3. Mittelhandknochen eingebracht wird ([7]; Abb. 3).

**Figure 4:**
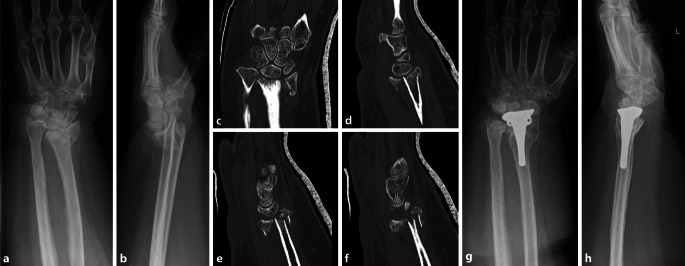

Eine weitere Möglichkeit ist die Versorgung mit einer Handgelenkhemiprothese analog zur Schenkelhals- oder zur proximalen Humerusfraktur, bei der ein Ersatz der distalen Radiusgelenkfläche, ggf. mit begleitender Darrach-Operation, erfolgt ([5, 13]; Abb. 4).

### Zusatzmaßnahmen

Persistiert eine Medianussymptomatik nach erfolgter gedeckter Reposition der Fraktur, ist die notfallmäßige Spaltung des Karpaltunnels im Rahmen der Frakturversorgung zur Vermeidung bleibender Nervenschäden indiziert. Eine prophylaktische Spaltung bei der Osteosynthese wird kontrovers diskutiert [6, 28] und von den Autoren des vorliegenden Beitrags nicht empfohlen.

Bei ausgeprägten zentralen metaphysären Trümmerzonen kann die Defektauffüllung mithilfe von autologem oder aber allogenem Knochentransplantat notwendig werden [24].

Nach erfolgter Osteosynthese sollte eine intraoperative Stabilitätstestung des distalen Radioulnargelenks (DRUG) in Neutral‑, Pro- und Supinationsstellung erfolgen. Auf weitere Begleitverletzungen und ihre Behandlung wird im Beitrag von Wieschollek und Megerle im gleichen Themenheft eingegangen.

### Wahl des geeigneten Osteosyntheseverfahrens in Abhängigkeit vom Frakturtyp – Entscheidungsfindung

Um die Auswahl des geeigneten Osteosyntheseverfahrens in Abhängigkeit vom Frakturtyp zu erleichtern, wird im Folgenden die interne Standard Operating Procedure (SOP) der Universitätsklinik für Orthopädie und Traumatologie, Medizinische Universität Innsbruck, erläutert. Diese orientiert sich an der Pechlaner-Klassifikation (Tab. 4; [25]). Eine individuelle Evaluation muss jedoch bei jeder Fraktur erfolgen, es können lediglich Richtlinien für die Behandlung formuliert werden.

**Table 4:** 

Typ	Beschreibung
I‑1	Dorsale metaphysäre Fraktur
I‑2	Dorsale metaphysär-artikuläre Fraktur
I‑3	Dorsale Luxationsfraktur
II‑1	Zentrale metaphysäre Fraktur
II-2A	Zentrale Impressionsfraktur
II-2B	Fraktur des Processus styloideus radii
II-2C	Ulnarer Randbruch
II-2D	Zentraler Mehrfragmentbruch
II‑3	Zentrale Luxationsfraktur
III‑1	Palmare metaphysäre Fraktur
III‑2	Palmare metaphysär-artikuläre Fraktur
III‑3	Palmare Luxationsfraktur

Ein Großteil der Frakturen ist mithilfe der isolierten palmaren winkelstabilen Plattenosteosynthese adäquat adressierbar. Dies gilt für dorsale metaphysäre Frakturen (Typ I-1), dorsale metaphysär-artikuläre Frakturen (Typ I-2), zentrale metaphysäre Frakturen (Typ II-1) und palmare metaphysäre Frakturen (Typ III-1).

**Figure 5:**
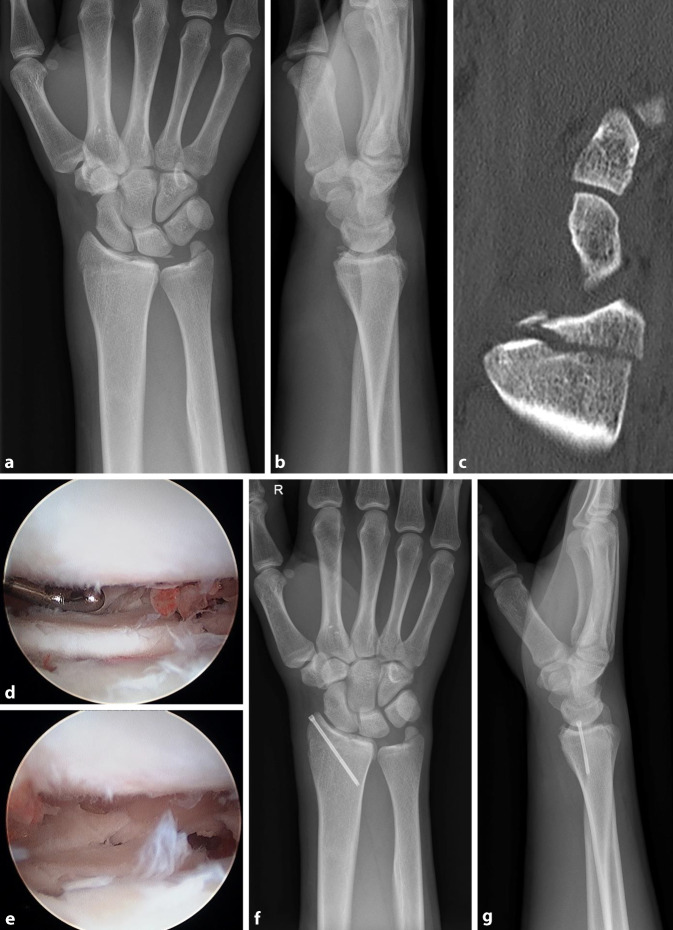

Die dorsale Luxationsfraktur (Typ I-3) wird mithilfe einer dorsalen Plattenosteosynthese versorgt. Bei zentralen Impressionsfrakturen (Typ II-2A) sollte zusätzlich zur palmaren Verplattung die Trümmerzone mit einem autogenen Knochentransplantat als vorderem Beckenkammspan, aufgefüllt werden. Begleitend erfolgt die Arthroskopie zur Evaluation der korrekten Fragmentreposition. Frakturen des Processus styloideus radii (Typ II-2B) sind der Osteosynthese mit kanülierten Schrauben zugänglich; zum Ausschluss einer Greater-Arch-Verletzung, die sehr häufig mit dieser Fraktur einhergeht, sollte eine zusätzliche Arthroskopie durchgeführt werden ([17]; Abb. 5).

Bei ulnaren Randbrüchen (Typ II-2C) muss zwischen palmarer und dorsaler Beteiligung der Fossa lunata unterschieden werden; Zugang und Versorgung erfolgen von der jeweils beteiligten Seite aus. Vor allem das palmare „lunate facet fragment“ ist aufgrund seiner Anatomie oft sehr schwer zu fixieren. Ein sekundärer Repositionsverlust ist schwer zu adressieren, führt zu inakzeptablen klinischen Ergebnissen und sollte unbedingt vermieden werden. Für die Fixation dieser palmaren ulnaren Fragmente haben sich speziell entwickelte Plattensysteme bewährt. Um das palmare Fragment für die Heilungsphase vom Druck zu entlasten, kann ein zusätzlicher radioulnarer K‑Draht von dorsal für 4 Wochen eingebracht werden.

**Figure 6:**
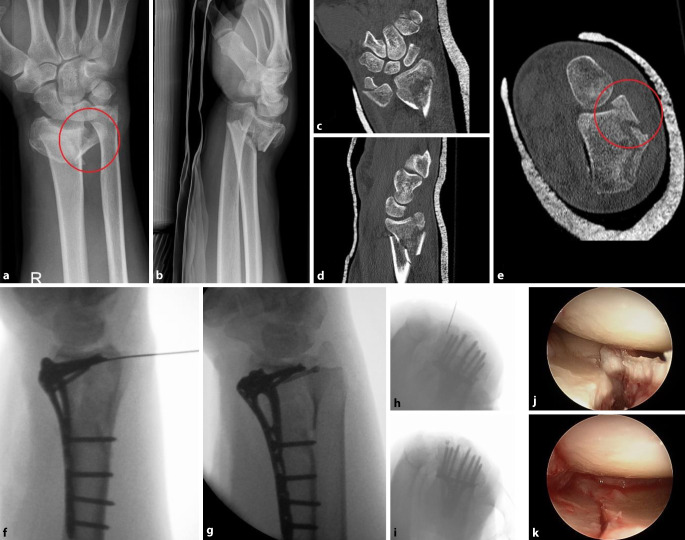

Beachtet werden müssen die korrekte Reposition und Fixation eines dorsalen Die-Punch-Fragments. Gerade diese Fragmente sind in der intraoperativen Röntgenkontrolle aufgrund von Überlagerungen der proximalen Handwurzelreihe sehr schwer zu kontrollieren. Eine intraartikuläre Stufe zwischen der dorsalen und palmaren Gelenkfläche stellt einen präarthrotischen Faktor dar. Gerade um die Reposition des dorsalen und ulnaren Kantenfragments zu kontrollieren, nutzen die Autoren des vorliegenden Beitrags die intraoperative Arthroskopie ([17, 21]; Abb. 6).

Eine intraartikuläre Stufe zwischen dorsaler und palmarer Gelenkfläche birgt ein Arthroserisiko

Die Versorgung zentraler Mehrfragmentbrüche (Typ II-2D) und zentraler Luxationsfrakturen (Typ II-3) gestaltet sich aufwendig. Um die optimale Reposition zu erreichen und einen sekundären Repositionsverlust zu verhindern, sollten das Repositionsergebnis arthroskopisch kontrolliert sowie bei Bedarf die metaphysäre Höhle mit einem autologen Knochentransplantats aufgefüllt und die Gelenkfläche unterstützt werden. Durch die Doppelplattenosteosynthese von palmar und dorsal gelingt meist eine suffiziente Fixation.

Für palmare metaphysär-artikuläre Frakturen (Typ III-2) sowie palmare Luxationsfrakturen (Typ III-3) bietet sich die arthroskopisch gestützte palmare Versorgung mit einer winkelstabilen Plattenosteosynthese an. Um auch weit distal gelegene Fragmente zu fassen, stehen spezielle Implantate wie z. B. Rand- oder Hakenplatten zur Verfügung.

### Postoperative Nachbehandlung

Prinzipiell sollte immer eine übungsstabile Osteosynthese erreicht werden, um eine frühfunktionelle Nachbehandlung zu ermöglichen. Trümmerfrakturen, Begleitverletzungen und fehlende PatientInnen-Compliance erfordern manchmal eine postoperative Immobilisierung. Eine Ruhigstellung in einer abnehmbaren Unterarmschiene bis zur 3. postoperativen Woche wird empfohlen, wobei jedoch sofortige aktive Beübungen der Fingergelenke und des Handgelenks, je nach Stabilität der Frakturversorgung, aus der Schiene heraus angestrebt werden sollen [34].

### Implantatentfernung

Im eigenen Kollektiv beobachten die Autoren Sehnenrupturen sowohl der Beuge- als auch Strecksehnen bis zu 10 Jahre postoperativ nach einer Plattenosteosynthese. Postoperativ asymptomatische Plattenpositionen können bei Zunahme der Beweglichkeit und Belastungbeginnen, mechanisch zu stören, und führen dann zu Symptomen. Da Sehnen keine Schmerzrezeptoren haben, werden über längerem Zeitverlauf bestehende Sehnenirritationen oft erst durch den Funktionsverlust bemerkbar [4].

Generell sollten sekundäre Sehnenrupturen durch mechanisch störende Implantate mit allen möglichen Maßnahmen vermieden werden. Bei jeglichem Verdacht einer Sehnenläsion führen die Autoren eine dynamische Ultraschalluntersuchung durch und können bei Bedarf früh reagieren.

Es wird empfohlen, Implantate, die dem Soong-Grad 2 entsprechen, nach der Frakturheilung so bald wie möglich zu entfernen [31]. Dorsale Platten können oft eine Flexionseinschränkung und Extensorsehnenirritationen verursachen. Bei Bedarf wird bei der Implantatentfernung der dorsalen Platte auch eine Teno- und Arthrolyse zur Verbesserung der Beweglichkeit durchgeführt.

## Fazit für die Praxis

- Distale Radiusfrakturen können die Funktionalität der Hand und die Lebensqualität der Betroffenen massiv beeinträchtigen.
- Die Wiederherstellung der anatomischen Verhältnisse ist unerlässlich.
- Offene Frakturen, Gefäß- und Nervenverletzungen, insbesondere mit traumatischem Karpaltunnelsyndrom, erfordern eine notfallmäßige Versorgung.
- Zur Diagnostik sollte neben der Durchführung von Anamnese, klinischer Untersuchung und Nativröntgen die Indikation zur CT-Bildgebung großzügig gestellt werden.
- Die adäquate PatientInnenaufklärung, einschließlich geplanter Nachbehandlung, ist essenziell.
- Aus der Vielzahl möglicher Verfahren muss entsprechend der Fraktur und dem PatienInnenanspruch das Bestmögliche gewählt werden.
- Die palmare winkelstabile Plattenosteosynthese hat sich in den meisten Fällen zur Methode der Wahl etabliert.
- Indikationen zur unterstützenden Arthroskopie sollten beachtet werden.
- Komplexe Frakturen können eine Kombination der beschriebenen Verfahren erfordern.
- Auf postoperative Komplikationen muss geachtet und entsprechend reagiert werden.

## Abkürzungen

CRIF: „Closed reduction and internal fixation“
CRPS: „Complex regional pain syndrome“
CT: Computertomographie
DRUG: Distales Radioulnargelenk
FPL: Musculus flexor pollicis longus
K‑Draht: Kirschner-Draht
ORIF: „Open reduction and internal fixation“
p.-a.: Posterior-anterior
pDMS: Periphere Durchblutung, Motorik und Sensibilität
SL: Skapholunär
TFCC: Triangulärer fibrokartilaginärer Komplex
